# Circumventing the “Ick” Factor: A Randomized Trial of the Effects of Omitting Affective Attitudes Questions to Increase Intention to Become an Organ Donor

**DOI:** 10.3389/fpsyg.2017.01443

**Published:** 2017-08-28

**Authors:** Sally Doherty, Elizabeth Dolan, Jennifer Flynn, Ronan E. O’Carroll, Frank Doyle

**Affiliations:** ^1^Department of Psychology, Division of Population Health Sciences, Royal College of Surgeons in Ireland Dublin, Ireland; ^2^Department of Psychology, Royal College of Surgeons in Ireland – Medical University of Bahrain Busaiteen, Bahrain; ^3^Division of Psychology, University of Stirling Stirling, Scotland

**Keywords:** organ donation, affective attitudes, question behavior effect, RCT

## Abstract

**Objectives:** Including or excluding certain questions about organ donation may influence peoples’ intention to donate. We investigated the effect of omitting certain affective attitudinal items on potential donors’ intention and behavior for donation.

**Design:** A cross-sectional survey with a subgroup nested randomized trial.

**Methods:** A total of 578 members of the public in four shopping centers were surveyed on their attitudes to organ donation. Non-donors (*n* = 349) were randomly assigned to one of three groups: Group 1 completed items on affective and cognitive attitudes, anticipated regret, intention, subjective norm and perceived behavioral control. Group 2 completed all items above but excluded affective attitudes. Group 3 completed all items but omitted negatively worded affective attitudes. The primary outcome was intention to donate, taking a donor card after the interview was a secondary behavioral outcome, and both were predicted using linear and logistic regression with group 1 as the reference.

**Results:** Mean (SD) 1–7 intention scores for groups 1, 2 and 3 were, respectively: 4.43 (SD 1.89), 4.95 (SD 1.64) and 4.88 (SD 1.81), with group 2 significantly higher than group 1 (β = 0.518, 95% confidence interval [CI] 0.18 to 0.86).At the end of the interview, people in group 2 (66.7%; OR = 1.40, 95% CI 0.94 to 2.07, *p* = 0.096) but not those in group 3 (61.7%; OR = 1.10, 95% CI 0.69 to 1.75, *p* = 0.685), were marginally more likely to accept a donor card from the interviewer than people in group 1 (59.7%).

**Conclusion:** Omitting affective attitudinal items results in higher intention to donate organs and marginally higher rates of acceptance of donor cards, which has important implications for future organ donation public health campaigns.

## Introduction

An organ transplant represents an opportunity for a new life for people with end-stage organ failure. Worldwide, 5-year survival rates are greater than 70% for all organs transplanted and are improving every year. Yet in the United Kingdom there are still three people dying every year due to a lack of available organs (Behavioural Insights Team, 2013; Death, 2013). Figures reported for 2014 in the European Union, Iceland, Norway and Turkey indicate a total of 86,000 people waiting for a transplant for a total population of 588 million (Eu-Commission, 2014).

While there appears to be a consensus that organ donation is acceptable to the public, there is still a paucity of available organs. United Kingdom and Irish figures indicate that while 90% of people are in agreement with donating organs, only one third of the public are registered on the United Kingdom database, or where there is no register, as in Ireland, similar proportions of the pubic carry organ donation cards (Healy et al., 2009; National Health Service [NHS], 2015). This gap in the supply and demand of organs has been described as a “chasm,” and much of the recent literature in this area has focused on how to increase organ donation (Satel and Cronin, 2015).

### Attitudes toward Organ Donation

The question of how to increase the supply of organs has been the focus of global medical, politic and social efforts. A recent study in the United States reported no observable effect from state initiatives to increase organ donation (Chatterjee et al., 2015). While attitudes are important predictors of organ donation, it may be important to consider the “balance between positive versus negative attitudes” (p. 646) (Morgan et al., 2008).

Particular research attention on improving intention to donate organs has focused on the role of affective attitudes. These are related to a person’s feelings or emotions, such as fear (the ‘jinx’ factor), or misunderstandings relating to procurement of organs, or discomfort when talking about death and disgust relating to medical procedures referred to as the “ick” factor (Morgan et al., 2008). United Kingdom research employing a previously validated affective attitude questionnaires devised by Morgan et al. (2008) has further investigated the role of affective attitudes. This United Kingdom research, employing an experimental manipulation of attitudes in relation to intention to register as posthumous donors reported the two attitudes related to disgust and bodily integrity, differentiated between those who signed up to organ donation registers and those who do not. Thus providing further evidence that visceral, affective attitudes act as barriers to registering for organ donation (O’Carroll et al., 2011b, 2012).

Other factors explored in the attempt to understand the barriers to organ donation include anticipated regret (AR). As people are motivated to avoid regret, promotion of AR should motivate people to undertake action to avoid future emotional consequences, in other words having higher AR over not donating organs should actually increase rates of donation. Previous research has found that AR based both on self-report (Richard et al., 1996) and behavior change interventions (Sandberg and Conner, 2009) has been found to be a reliable indicator of an action being undertaken (Koch, 2014; Brewer et al., 2016). O’Carroll et al. (2011b) found that inclusion of two AR questions to an affective attitudes questionnaire increased self-reported organ donation intentions. In a subsequent pilot study, organ donor registration for those randomized to the AR group was reported by almost 22% compared to 13% of the control group and 8.5% of a theory of planned behavior group (O’Carroll et al., 2011a). These results suggested a promising avenue for overcoming affective attitudes relating to the “ick” factor and bodily integrity and increasing organ donation. However, the results relied on self-report data, and actual behavior may differ.

In order to determine if this finding could be replicated for verified organ donor registration, indicated by joining a national organ donor registry, the methods were replicated in a randomized trial of 14,509 Scottish adults. This trial assigned people to one of four groups: No Questionnaire Control (NQC), Questionnaire Control (QC), including items on affective attitude and intention, Theory of Planned Behavior (TPB: affective attitude and TPB items), Anticipated Regret (AR: affective attitude, TPB and AR items). The study sought to assess an increase in verified organ donation 6 months after the study (O’Carroll et al., 2012). However, contrary to their previous findings, verified organ donor registration was *lower* in the AR group compared to the NQC group, and the NQC group actually had the highest level of registration (O’Carroll et al., 2016). The authors speculated that people exposed to affective attitude items may have been primed or cued to think more negatively about organ donation and this may have counteracted any positive effect of AR.

There are, however, other possible explanation of this finding. The first relates to the content of the questionnaires, known as the mere measurement effect; which proposes that by merely asking the question, using either positively or negatively worded items, increases the accessibility of attitudes and impacts on contextual cues (Wood et al., 2014). The impact of contextual cues has previously been explored in various studies related to blood donation and inclusion of specific cues relating to HIV, which were perceived as negative and this decreased participation in blood donation (Farell et al., 2002). Whereas the positive effect of increasing intention and/or behavior of contextual cues with the inclusion of AR items in a questionnaire was found to significantly increase attendance at cervical screening clinics (Sandberg and Conner, 2009). This would suggest that depending on the positive or negative perception of the question, behavior and/or intention will increase or decrease. A recent meta-analysis suggests that the Question Behaviour Effect (QBE) mediates the relationship between intention and behavior, proposing that intention to behave increases when related to a socially desirable behavior and has no significant effect on undesirable behaviors(Wilding et al., 2016). A second possibility is that that previous research activated both cognitive and affective attitudes simultaneously, which may have masked any potential independent effects, although this has not been investigated in the organ donation literature.

### The Present Study

Further investigation of affective attitudes to organ donation is important to inform future interventional campaigns. The present study therefore replicates previously used methodologies (Morgan et al., 2008; O’Carroll et al., 2011b) to:

(1) Investigate attitudes to organ donation in Ireland.
(2) Experimentally manipulate the presence of positive or negative affective attitudes to see if including or omitting these effects intention to donate or accept a donor card. We hypothesized that omitting negatively worded items would be associated with a higher intention to become an organ donor, and higher rates of organ donor behavior (i.e., taking an organ donor card).

### The Research Question

Does including or omitting affective attitude items effect intention to donate or acceptance of an organ donor card in non-donors?

## Materials and Methods

### Context

Ireland does not have a register for organ donors. Willingness to donate can be indicated in a number of ways: (1) carrying a signed organ donor card, (2) indicating donation status on drivers license, (3) providing information on an organ donation app, and (4) discussing one’s wishes with family members. Next-of-kin can veto the donation of organs, even if a person completes any of 1–3 above. Therefore, we defined *willingness to donate* as meeting any of the above 4 conditions, and an *organ donor* as someone who had met any of 1–3 *and* the critical 4th condition. *Non-donors* are therefore defined as someone who has not met either conditions 1–3 *and* discussed their wishes with their family (condition 4).

#### Design

A cross sectional survey with subgroup nested single-blind randomized trial.

#### Outcomes

The primary outcome was intention to donate. Taking a donor card after the interview was a secondary behavioral outcome.

### Participants

The protocol was approved by the Royal College of Surgeons in Ireland Research Ethics Committee (REC; reference number REC1048). An opportunistic sample of members of the public, aged 18-years-old and over, were recruited by four medical school students in four large shopping centers. Shoppers were approached by students and asked to participate in the study. Those who agreed provided their information relating to gender, age, health insurance status and attitudes to organ donation. Two students conducted the majority of the interviews (286 and 266, respectively) while two other students interviewed the remaining 26 participants. Data was collected with Survey Monkey on iPads. All participants were provided with a participant information leaflet and verbally consented to participate. Signed consent was not required by the REC as the survey was anonymous – no identifying data was obtained from participants.

### Procedure

Participants were randomly assigned by a computer generation program to one of three groups:

Group 1: Replication group – this group completed the entire questionnaire, in order to replicate the findings of O’Carroll et al. (2011a).
Group 2: Omitting affective attitudes – this group completed the questionnaire, but 16 affective attitude items were omitted.
Group 3: Omitting negatively worded affective attitudes – this group completed the questionnaire, but 12 negatively worded affective attitude items were omitted.

### Measures

As mentioned above, we used a modified version of the attitudes to organ donation questionnaire (Morgan et al., 2008; O’Carroll et al., 2011b, 2012) with minor changes to the wording that reflect the status of organ donation in Ireland. For non-donors, items replaced the word ‘register’ with ‘sign up for organ donation and discuss this with my family.’ For example, an intention item was reworded as follows: “I will definitely sign up for organ donation and discuss this with my family in the next few months.”

*Affective attitudes* was measured by 16 items on a 7-point scale (1 = strongly disagree to 7 = strongly agree). The items include components of beliefs associated with organ donation. (See Appendix 1 for affective attitude items and group inclusion).

*Perceived benefits* was measured by four items (e.g., “organ donation helps to bring meaning to the death of a loved one”).

*Bodily integrity* was measured by two items (e.g., “removing organs from the body just isn’t right”).

*Medical distrust* was measured by four items, (e.g., “sometimes medical procedures are done on people without their consent”).

*Ick factor* was measured by three items (e.g., “the idea of organ donation is somewhat disgusting”).

*Jinx factor* was measured by three items, (e.g., “people who donate their organs risk displeasing God or nature”).

*Anticipated regret* was measured by two items, (e.g., “if I did not sign up for organ donation in the next few months, I would later wish I had”).

*Cognitive attitudes* was measured by three items (e.g., “I view organ donation as a benefit to humanity”).

*Perceived behavioral control* was measured by two items (e.g., “how much control do you have over signing up for organ donation in the next few months?”).

*Intention* was measured by two items (e.g., “how strong is your intention to sign up for organ donation in the next few months?”). Intention to donate was the primary outcome of interest.

*Subjective norm* was measured by two items (e.g., people who are important to me think I should sign up for organ donation and confirm this with my family in the next few months”).

At the end of the interview, interviewers also recorded whether participants were offered an organ donation card, and whether they accepted it. Accepting the donor card was the secondary outcome of interest.

### Statistical Analysis

We used χ^2^, *t*-test and analysis of variance as appropriate for descriptive analyses. As the survey was conducted in four different shopping centers, which serve different demographic and socioeconomic populations, participants in a given shopping center are likely to have more similar characteristics (e.g., higher prevalence of those with private health insurance in higher socioeconomic areas) than people randomly sampled from the general population. This can lead to errors in estimated standard errors and confidence intervals. The ‘cluster’ option in Stata was therefore used to provide Huber-White robust variance estimators to account for this clustering in all subsequent analyses (Rogers, 1993). Logistic regression was used to test the association between psychological variables and willingness to donate organs. Then, for hypothesis testing for non-donors, we used linear regression, with group 1 (all questionnaire items) as the reference group. We also determined whether the responses were associated with the interviewer (omitting those who conducted a small number of interviews – i.e., 26 participants), and adjusted for this or other significant demographic predictors of organ donation status in subsequent models. Logistic regression was used to predict any differences in interviewers offering a donor card to participants. Logistic regression was then used to predict whether participants accepted a donor card or not, when adjusting for the interviewer and other relevant factors as above. The *margins* command in Stata provided the adjusted prevalence rates for those accepting the donor card. Finally, we conducted a *post hoc* analysis where we combined omitted affective attitudes condition versus the other two combined for accepting an organ donor card.

## Results

### Sample Description

**FIGURE 1:**
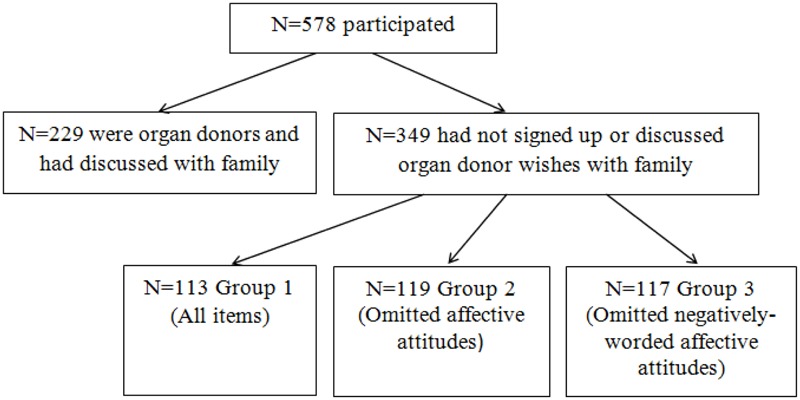
Participant flow-chart.

The participant flow-chart is shown in **Figure 1** below. A total of 578 people participated in the survey, with 349 being defined as non-donors.

Regarding organ donor status, 169/578 (29%) reported carrying an organ donor card, 4/576 (0.69%) reported having the organ donor app, 160/545 (29.4%) reported having organ donor status recorded on their driving license. The majority 315/578 (54.5%) reported having discussed their wishes with their family. Overall, this left 349/578 (60.4%) non-donors.

Descriptive statistics for the sample and subsamples are available in **Table 1** below. Shopping center location was associated with donor status – the proportion of donors varied significantly by shopping center, with the proportion of donors ranging from 55.9% (centers 1 and 4, 76/136 each) to 68.3% (138/202, center 3), with center 2 yielding 56.7% (59/104) donors. Donors were also more likely to have given blood in the past, and also more likely to know others who had donated an organ or who needed an organ. No significant differences among the experimental groups were observed, indicating that randomisation was successful.

**Table 1:** Sample description.

	Total	Donors	Non-	Statistic	*p*-value	Group 1	Group 2	Group 3	Statistic	*P*-value
	sample	(*n* = 229)	donors			(*n* = 113)	(*n* = 119)	(*n* = 117)		
	(*n* = 578)		(*n* = 349)							
Age (mean, SD, *n* = 576)	41.2 (16.4)	41.1 (14.9)	41.3 (17.4)	*t* = -0.12	0.901	40.2 (17.8)	42.8 (16.8)	40.6 (17.5)	*F* = 0.73, *df* = 2	0.482
Women	54.9%	57%	53.5%	χ^2^= 0.709	0.400	46.4%	44.5%	48.7%	χ^2^= 0.42	0.812
Insurance										
Private	54.1%	54.2%	54%	χ^2^= 1.85	0.396	58%	57.1%	47%	χ^2^= 5.29	0.259
None/Public	31%	33.2%	29.6%			28.6%	24.4%	35.9%		
Medical Card	14.9%	12.6%	16.4%			13.4%	18.5%	17.1%		
Shopping Center (anonymised)										
1	23.5%	26.2%	21.8%	χ^2^= 8.20	0.042^∗^	23.9%	22.7%	18.8%	χ^2^= 2.43	0.876
2	18%	19.7%	16.9%			17.7%	15.1%	18%		
3	35%	28%	39.5%			35.4%	39.5%	43.6%		
4	23.5%	26.2%	21.8%			23%	22.7%	19.6%		
Do you know someone who… (% yes)										
has received an organ	33.2%	37.6%	30.4%	χ^2^= 3.22	0.073	31%	32.8%	27.3%	χ^2^= 0.849	0.654
needs an organ	17.1%	21%	14.6%	χ^2^= 3.92	0.048^∗^	11.5%	16.8%	15.4%	χ^2^= 1.39	0.499
has donated an organ	20.8%	26.6%	17%	χ^2^= 7.86	0.005^∗∗^					
Ever donated an organ	0.17%	–	–	–	–	–	–	–	–	–
Ever donated blood	41.7%	57.3%	31.6%	χ^2^= 37.2	<0.001^∗∗∗^	37.2%	28.6%	29.3%	χ^2^= 2.41	0.300

*^∗^*p* < 0.05, ^∗∗^*p* < 0.01, ^∗∗∗^*p* < 0.001*

### Predicting Organ Donor Status

The logistic regression models predicting willingness to donate are shown in **Table 2**. Higher scores on AR and subjective norms increased the probability of being willing to donate to a statistically significant degree. Higher scores on bodily integrity, medical distrust, and the “ick” and jinx factors were associated with reduced probability of willingness to donate.

**Table 2:** Logistic regression models predicting willingness to donate.

Univariate	OR	95% CI	*p*
Perceived benefits (*n* = 372)	1.13	0.96 to 1.34	0.140
Bodily integrity (*n* = 194)	0.70	0.50 to 0.97	0.034^∗^
Medical distrust (*n* = 194)	0.70	0.62 to 0.80	<0.001^∗∗∗^
Ick factor (*n* = 194)	0.50	0.36 to 0.69	<0.001^∗∗∗^
Jinx factor (*n* = 193)	0.49	0.31 to 0.77	0.002^∗∗^
Anticipated regret (*n* = 343)	1.27	1.10 to 1.46	0.001^∗∗^
Cognitive attitude (*n* = 341)	1.12	0.84 to 1.51	0.437
Subjective norm (*n* = 349)	1.42	1.24 to 1.63	<0.001^∗∗∗^
Perceived behavioral control (*n* = 348)	1.14	0.81 to 1.60	0.467

*^∗^*p* < 0.05, ^∗∗^*p* < 0.01, ^∗∗∗^*p* < 0.001.*

### Predicting Intention

**FIGURE 2:**
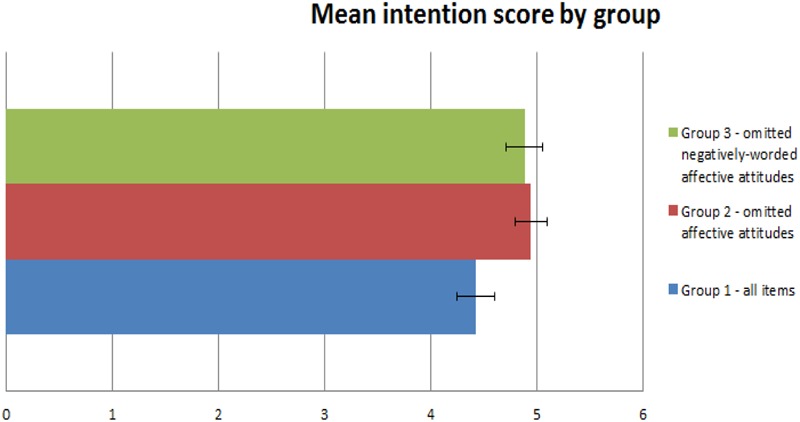
Mean intention to donate scores.

Intention to donate scores in non-donors were available for 112, 113 and 116 participants in groups 1, 2 and 3, respectively shown in **Figure 2** below. Mean (SD) scores for each group in turn were: 4.43 (SD 1.89), 4.95 (SD 1.64) and 4.88 (SD 1.81). The linear regression models are displayed in **Table 3**. Group 2 had the highest mean intention score, which was significantly different to Group 1, even when adjusting for other potentially important factors in the different models. Although Group 3 had a marginally larger mean intention score to Group 1, this did not reach statistical significance in any of the adjusted models.

**Table 3:** Linear regression models assessing group effects predicting intention to donate.

	Intention	β	Std. Err.	95% CI	*P*
Model 1 (*n* = 341)	Group 1	Ref.	–	–	–
	Group 2	0.518	0.11	0.18 to 0.86	0.017^∗^
	Group 3	0.455	0.22	-0.26 to 1.17	0.136
Model 2 (*n* = 318)	Group 1	Ref.	–	–	–
	Group 2	0.552	0.13	0.14 to 0.97	0.024^∗^
	Group 3	0.486	0.20	-0.14 to 1.12	0.091
	Researcher	-0.522	0.13	-0.95 to -0.10	0.29^∗^
Model 3 (*n* = 317)	Group 1	Ref.	–	–	–
	Group 2	0.59	0.17	0.05 to 1.12	0.039^∗^
	Group 3	0.49	0.18	-0.09 to 1.07	0.074
	Researcher	-0.39	0.14	-0.85 to 0.67	0.073
	Know anyone who has received blood	-0.11	0.20	-0.74 to 0.52	0.619
	Needs an organ donation	0.63	0.24	-0.13 to 1.38	0.077
	Has donated an organ	-0.02	0.27	-0.88 to 0.83	0.933
	Ever donated blood	0.54	0.24	-0.22 to 1.29	0.110

*^∗^*p* < 0.05, ^∗∗^*p* < 0.01, ^∗∗∗^*p* < 0.001.*

### Predicting Behavior

A donor card was accepted by 48% of participants overall, of which 63% were non-donors. There was a significant difference between the two main researchers in terms of offering a card (OR = 0.33, 95% CI 0.25 to 0.43, *p* < 0.001 for the experimental groups, OR = 0.08, 95% CI 0.06 to 0.12, *p* < 0.001 for the donors and non-donors combined), therefore further analysis adjusted for the researcher. At the end of the interview, and when analyzing the entire sample including donors and non-donors, people in groups 2 (49.7%; OR = 1.47, 95% CI 1.11 to 1.95, *p* = 0.007) and 3 (47.8%; OR = 1.33, 95% CI 1.02 to 1.75, *p* = 0.035) were more likely to accept a donor card from the interviewer than people in group 1 (42.4%), when adjusting for the interviewer. When analyzing non-donors only, those in group 2 were marginally more likely to accept a card (66.7%; OR = 1.40, 95% CI 0.94 to 2.07, *p* = 0.096) but those in group 3 were not (61.7%; OR = 1.10, 95% CI 0.69 to 1.75, *p* = 0.685), when comparing to those in group 1 (59.7%). When combining groups 1 and 3 for *post hoc* analysis, non-donors in group 2 were more likely to accept a card than were the combination of groups 1 and 3 (OR = 1.33, 95% CI 1.09 to 1.63, *p* = 0.006). Adding intention to the original logistic models meant that groups were no longer significant predictors of donor card acceptance. Higher intention scores were associated with significantly higher likelihood of acceptance of a donor card at the end of the interview (OR = 2.02, 95% CI 1.78 to 2.29, *p* < 0.001), and adjusting for group did not affect this finding (OR = 2.04, 95% CI 1.79 to 2.32, *p* < 0.001).

## Discussion

This study confirms the importance of emotional factors in predicting organ donor status. We demonstrated that omitting affective attitudinal items lead to higher intention to donate, and marginally higher rates of acceptance of donor cards, among non-donors. We also demonstrated that in the overall sample of donors and non-donors, and when combining data from groups 1 and 3 in *post hoc* analysis, omitting attitudinal items led to higher numbers of people accepting donor cards from interviewers. The results suggest, in the context of organ donation, that omitting reference to affective attitudes when using an AR intervention may increase intention to donate, and ultimately rates of donation, although this would have to be confirmed in future research using verified organ donor registration behavior as the primary outcome.

These results are important as they may go some way toward explaining the recent disappointing findings outlined previously, where including affective attitudes in three arms of a 4-arm trial resulted in higher rates of donor registration in the NQC arm, (i.e., in those who did not receive any questions on affective attitudes) (O’Carroll et al., 2012, 2016). The authors speculated that questionnaire item content may have primed negative perceptions of organ donation in a contextual cueing effect, and the present findings support this hypothesis.

Priming or contextual cues have been shown to influence the judgements made by research participants (Strack et al., 1988). A previous public survey examined questions which were cued relating to risk of HIV and blood donation. Results indicated that respondents were 11 times more likely to respond “yes that the virus can be contracted by donating blood” to a survey question about blood donation and HIV transmission compared to the group who had not been informed about the risk of HIV in blood transfusions. The authors suggest that contextual cues within the survey methodology had an impact on the survey outcome based on the HIV concept being more readily available to the participants, who may well have not otherwise considered the risks (Farell et al., 2002). This could also be the case in relation to asking questions related to organ donation and cueing negative attitudes to bodily integrity, disgust (ick) and the jinx factor.

Another similar explanation to priming or contextual cueing for our results could be related to message framing, wherein health information is presented as a gain or a loss and the perception of the health risk is associated with subsequent behavior (Bartels et al., 2010; McGregor et al., 2012). In the current study, organ donation was primed by a series of attitudinal questions mostly relating to aversive consequences, which may have prompted respondents to view their decision to take a donor card as a loss and subsequently not take a card based on their perceived assessment of risk associated with organ donation.

Previous research on the QBE suggests that eliciting positive or negative attitudes has an impact on subsequent behavior (Godin et al., 2008; Wilding et al., 2016). The findings from the current study provide evidence for omitting both positive and negative attitudinal items from an organ donation questionnaire. This omission subsequently led to an increase in behavior to take an organ donation card. Organ donation could be perceived as an emotionally negative or difficult subject, but by omitting emotive factors the participant is not primed to elicit feelings and makes a decision to behave based on the other components of the questionnaire, which in this instance focused on cognition, perceived behavioral control and subjective norms. Given that previous research has also investigated both cognitive and affective attitudes simultaneously, the present study is also novel in that these were separated experimentally, with the groups where all affective attitudes omitted had the best outcomes.

## Limitations and Strengths

This study has several limitations. Self-reported behavioral intention was measured as the primary outcome instead of actual registration behavior. Information on actual registration behavior is not available since Ireland does not have a donor registry; although a good indication of intention was captured by those taking a donor card. This situation also necessitated minor wording changes in the questionnaire, although it is unclear if such changes could have an effect on the results – future research should determine if reliability of items change significantly with such adjustments. No *a priori* power calculation was conducted, so students recruited participants for the full duration of a summer research placement. The present results will allow sample size calculations for future similar research. The sample this was large enough to find a difference in the primary outcome, but not the secondary outcome. The present results will allow sample size calculations for future similar research. Recruitment was not blinded – by necessity interviewers knew to which group the next person they approached was randomized – thus the results represent those from a single-blind trial, which could have biased the results. However, significant differences between recruiters did not impact on the main results. The sampling methods mean that the results are not generalizable to the Irish population. Another possible explanation and a limitation for our findings was the shorter questionnaire for participants in groups 2 and 3. O’Carroll et al. (2016) used filler items to ensure the same number of items in each arm. This may have had an impact on the outcome of our survey. Future research such as a randomized trial with an objective outcome measure of registration for organ dononation could be conducted replicating the current study using filler items to ensure all non-donors had equivalent length questionnaires.

Strengths include the adoption of established methodologies and items from previous research, and the recruitment of participants from four shopping centers, providing a level of robustness to the findings. Another strength is the measurement of both intention and behavior, with somewhat similar findings for both.

## Conclusion

Inclusion of affective attitude items in relation to organ donation would appear to undermine intention to become an organ donor. Our study suggests that questions relating to affective attitudes should be carefully considered, and probably omitted, in public health campaigns attempting to persuade the public to donate their organs.

## Author Contributions

SD and FD designed the protocol. SD drafted the paper and FD conducted the statistical analysis. ED and JF collected all data. RO kindly contributed his expertise in this field.

## Conflict of Interest Statement

The authors declare that the research was conducted in the absence of any commercial or financial relationships that could be construed as a potential conflict of interest.
