# Supplementary material for: Circumventing the “Ick” Factor: A Randomized Trial of the Effects of Omitting Affective Attitudes Questions to Increase Intention to Become an Organ Donor
Source: Front Psychol. 2017 Aug 28;8:1443. doi: 10.3389/fpsyg.2017.01443 (PMC5581398; doi:10.3389/fpsyg.2017.01443)
Supplement: Supplementary file 1 [file Table_1.DOCX]

| Affective attitudes: 16 items recorded on a seven point Likert scale | | | | | | |
| --- | --- | --- | --- | --- | --- | --- |
| **#** | **Item** | **Sub scale** | **Aspect** | **Group 1**  ***Completed All AA questions*** | **Group 2**  ***Omitted all AA questions*** | **Group 3**  ***Completed positive AA questions*** |
| 1 | Organ donation allows something positive to come out of a person’s death | Benefits | Positive | x |  | x |
| 2 | Organ donation helps to bring meaning to the death of a loved one | Benefits | Positive | x |  | x |
| 3 | Removing organs from the body just isn’t right | Integrity | Negative | x |  |  |
| 4 | The body should be kept whole for burial | Integrity | Negative | x |  |  |
| 5 | Hospitals sometimes prescribe medication as a way of experimenting on people without their knowledge or consent | Distrust | Negative | x |  |  |
| 6 | If I sign an organ donor card, doctors might take my organs before I’m actually dead | Distrust | Negative | x |  |  |
| 7 | Sometimes, medical procedures are done on people without their consent | Distrust | Negative | x |  |  |
| 8 | If I sign an organ donor card, doctors might not try so hard to save my life | Distrust | Negative | x |  |  |
| 9 | The idea of organ donation is somewhat disgusting | Ick factor | Negative | x |  |  |
| 10 | I wouldn’t like the idea of having another person’s organs inside of me, even if I needed an organ transplant | Ick factor | Negative | x |  |  |
| 11 | The thought of organ donation makes me uncomfortable | Ick factor | Negative | x |  |  |
| 12 | People who donate their organs risk displeasing God or nature | Jinx factor | Negative | x |  |  |
| 13 | The surest way to bring about my own death is to make plans for it like signing an organ donor card | Jinx factor | Negative | x |  |  |
| 14 | Organ donors may not be resurrected because they don’t have all their ‘parts’ | Jinx factor | Negative | x |  |  |
| 15 | Organ donors are heroic because they save lives | Benefits | Positive | x |  | x |
| 16 | Donating organs would allow part of me to live after I die | Benefits | Positive | x |  | x |
