# Supplementary material for: Circumventing the “Ick” Factor: A Randomized Trial of the Effects of Omitting Affective Attitudes Questions to Increase Intention to Become an Organ Donor
Source: Front Psychol. 2017 Aug 28;8:1443. doi: 10.3389/fpsyg.2017.01443 (PMC5581398; doi:10.3389/fpsyg.2017.01443)
Supplement: Supplementary file 2 [file Table_2.PDF]

Affective attitudes: 16 items recorded on a seven point Likert scale						
**#**	**Item**	**Sub scale**	**Aspect**	**Group 1** *Completed All AA questions*	**Group 2** *Omitted All AA questions*	**Group 3** *Completed positive AA questions*
1	Organ donation allows something positive to come out of a person's death	Benefits	Positive	X		X
2	Organ donation helps to bring meaning to the death of a loved one	Benefits	Positive	X		X
3	Removing organs from the body just isn't right	Integrity	Negative	X		
4	The body should be kept whole for burial	Integrity	Negative	X		
5	Hospitals sometimes prescribe medication as a way of experimenting on people without their knowledge or consent	Distrust	Negative	X		
6	If I sign an organ donor card, doctors might take my organs before I'm actually dead	Distrust	Negative	X		
7	Sometimes, medical procedures are done on people without their consent	Distrust	Negative	X		
8	If I sign an organ donor card, doctors might not try so hard to save my life	Distrust	Negative	X		
9	The idea of organ donation is somewhat disgusting	Ick factor	Negative	X		
10	I wouldn't like the idea of having another person's organs inside of me, even if I needed an organ transplant	Ick factor	Negative	X		

11	The thought of organ donation makes me uncomfortable	Ick factor	Negative	X		
12	People who donate their organs risk displeasing God or nature	Jinx factor	Negative	X		
13	The surest way to bring about my own death is to make plans for it like signing an organ donor card	Jinx factor	Negative	X		
14	Organ donors may not be resurrected because they don't have all their 'parts'	Jinx factor	Negative	X		
15	Organ donors are heroic because they save lives	Benefits	Positive	X		X
16	Donating organs would allow part of me to live after I die	Benefits	Positive	X		X
